# Abiraterone induces *SLCO1B3* expression in prostate cancer via microRNA-579-3p

**DOI:** 10.1038/s41598-021-90143-4

**Published:** 2021-05-24

**Authors:** Roberto H. Barbier, Edel M. McCrea, Kristi Y. Lee, Jonathan D. Strope, Emily N. Risdon, Douglas K. Price, Cindy H. Chau, William D. Figg

**Affiliations:** 1 Molecular Pharmacology Section, Genitourinary Malignancies Branch, Center for Cancer Research, National Cancer Institute, National Institutes of Health, 9000 Rockville Pike, Building 10, Room 5A03, Bethesda, MD 20892 USA; 2 Clinical Pharmacology Program, Center for Cancer Research, National Cancer Institute, National Institutes of Health, Bethesda, MD 20892 USA

**Keywords:** Cancer, Cancer therapeutic resistance

## Abstract

Understanding mechanisms of resistance to abiraterone, one of the primary drugs approved for the treatment of castration resistant prostate cancer, remains a priority. The organic anion polypeptide 1B3 (OATP1B3, encoded by *SLCO1B3*) transporter has been shown to transport androgens into prostate cancer cells. In this study we observed and investigated the mechanism of induction of *SLCO1B3* by abiraterone. Prostate cancer cells (22Rv1, LNCaP, and VCAP) were treated with anti-androgens and assessed for *SLCO1B3* expression by qPCR analysis. Abiraterone treatment increased *SLCO1B3* expression in 22Rv1 cells in vitro and in the 22Rv1 xenograft model in vivo. MicroRNA profiling of abiraterone-treated 22Rv1 cells was performed using a NanoString nCounter miRNA panel followed by miRNA target prediction. TargetScan and miRanda prediction tools identified hsa-miR-579-3p as binding to the 3′-untranslated region (3′UTR) of the *SLCO1B3*. Using dual luciferase reporter assays, we verified that hsa-miR-579-3p indeed binds to the *SLCO1B3* 3′UTR and significantly inhibited *SLCO1B3* reporter activity. Treatment with abiraterone significantly downregulated hsa-miR-579-3p, indicating its potential role in upregulating *SLCO1B3* expression. In this study, we demonstrated a novel miRNA-mediated mechanism of abiraterone-induced *SLCO1B3* expression, a transporter that is also responsible for driving androgen deprivation therapy resistance. Understanding mechanisms of abiraterone resistance mediated via differential miRNA expression will assist in the identification of potential miRNA biomarkers of treatment resistance and the development of future therapeutics.

## Introduction

Androgens and androgen receptor (AR) signaling drive prostate carcinogenesis. While androgen ablation therapy (ADT) has been the cornerstone of treatment for advanced prostate cancer, patients inevitably progress to castrate-resistant prostate cancer (CRPC) despite castrate levels of androgens (serum testosterone < 50 ng/mL). The current treatment armamentarium includes androgen biosynthesis inhibitors (e.g., abiraterone) and AR antagonists (e.g., enzalutamide, apalutamide, and darolutamide) that target persistent androgen production and AR signaling. Although these drugs prolong survival in men with CRPC, resistance eventually ensues; therefore, understanding mechanisms of resistance will facilitate future drug discovery and development.

Multiple groups have demonstrated *SLCO1B3* induction in cancerous versus healthy tissue^1,2^. Our laboratory and others have investigated the role of steroid hormone transporters in modulating intratumoral androgen concentrations that promote CRPC progression^1–5^. We showed that the organic anion polypeptide 1B3 (OATP1B3) transporter facilitates the diffusion of unconjugated testosterone into cells, and polymorphic variations in the *SLCO1B3* gene (encoding OATP1B3) are related to both the progression-free survival of men receiving ADT for hormone-responsive prostate cancer and the overall survival of men with CRPC^3,4^. We subsequently showed de novo OATP1B3 expression in prostate tumor cells contributes to greater androgen uptake, which is consistent with its role in disease progression^1^.

Because OATP1B3 is an important predictive marker in men with prostate cancer, it is essential to understand mechanisms governing its de novo expression. Transcriptional regulatory mechanisms for *SLCO1B3* expression include the involvement of different transcription factors and is highly dependent on tissue types. The *SLCO1B3* promoter can be transactivated by the farneosid X receptor (FXR)^6^, hepatic nuclear factor-1α^7^, and STAT5^6^, while the hepatocyte nuclear factor 3β^8^, and constitutive androstane receptor^9^ have been reported to repress transcription. Hypoxia-mediated^10,11^ and epigenetic mechanisms such as DNA methylation^10,12–14^ can also play a significant role in *SLCO1B3* mRNA expression.

The transcriptional regulation of *SLCO1B3* in prostatic tissue has not been fully elucidated. We have recently discovered that chetomin is a potent inducer of *SLCO1B3* transcription in prostate cancer cells^1^. Chetomin targets p300 (E1A binding protein), a global transcriptional coactivator, by disrupting the structure of its CH1 domain and precludes its interaction with the hypoxia-inducible factor-1*α* (HIF-1*α*) transcription factor. We found that modulation of HIF-1α only modestly increased *SLCO1B3* expression, suggesting that expression of *SLCO1B3* may occur via a p300-mediated regulatory mechanism^1^. Previous studies have shown that p300 increases during ADT and that this upregulation is associated with increased tumor growth and progression; conversely, increasing androgens produced a dose-dependent decrease in p300 expression^15^.

MicroRNAs (miRNAs) are small noncoding RNA molecules that function as key post-transcriptional regulators of gene expression by promoting mRNA degradation or translational repression^16^. The regulation of drug transporters by miRNAs has recently been investigated for the human intestine^17^. Though little research has involved miRNA in the direct regulation of *SLCO1B3* in prostate cancer, *SLCO1B3* was found to be negatively correlated with rifampin-induced miRNA expression in hepatocytes^18^. In addition, FXR and its target genes including *SLCO1B3* are regulated by hsa-miR-192, also in hepatocytes^19^. More broadly, miRNA involvement in prostate cancer initiation, proliferation, progression, and therapeutic resistance has been relatively well described, with thousands of relevant miRNA species characterized. miRNAs have been found to regulate key driving pathways in prostate cancer, such as the AR signaling axis, TMPRSS2-ERG, and PTEN^20–23^.

In this study, we investigated the effect of the second-generation anti-androgen abiraterone on *SLCO1B3* expression. We found that abiraterone stimulated the expression of *SLCO1B3* and the mechanism of this effect involved regulation by miRNAs.

## Results

### Abiraterone treatment and increased *SLCO1B3* expression in prostate cancer cells

**Figure 1:**
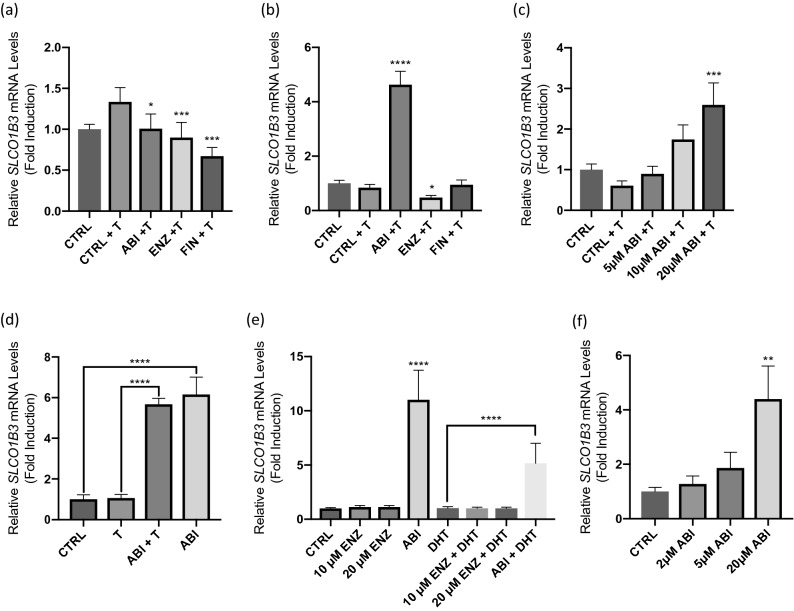
AR-positive prostate cancer cell lines (LNCaP, 22Rv1, and VCaP) were treated with 1 nM testosterone and three prostate cancer drugs abiraterone (ABI), enzalutamide (ENZ), or finasteride (FIN) for 24 h. RNA was extracted and assessed for changes in *SLCO1B3* expression using qPCR. Gene expression was normalized to expression of β-actin. (**a**) All treatment groups produced a significant reduction in its expression at 72 h in LNCaP Cells. (**b**) Abiraterone produced a highly significant upregulation of *SLCO1B3* in 22Rv1 cells. (**c**) Concentration-dependent effect of abiraterone (ABI) significantly increased the expression of *SLCO1B3.* 22Rv1 cells were treated in phenol red-free, charcoal-stripped media with abiraterone (ABI), enzalutamide (ENZ) in the presence or absence of (**d**) 1 nM testosterone (T) or (**e**) 1 nM dihydrotestosterone (DHT) for 24 h. 22Rv1 cells were also treated with a higher dose (20 μM) of ENZ to confirm that ENZ treatment has no effect on *SLCO1B3* expression. (**f**) The AR-positive prostate cancer cell line, VCaP, was treated with abiraterone at increasing concentrations for 24 h. ABI increased SLCO1B3 in a dose-dependent manner. RNA was harvested from cells after 24 h of treatment and assessed for changes in *SLCO1B3* expression using qPCR analysis. Gene expression was normalized to expression of β-actin. Treatment in AR-negative PC3 cells did not affect *SLCO1B3* expression (data not shown). Statistical tests were performed against CRTL + T for A-C. These data are the result of three independent experiments. *P < 0.05, **P < 0.001, ***P < 0.0001, ****P < 0.00001.

The relationship between p300/CBP and ADT has been characterized in advanced prostate cancer^15,24^. Since p300-mediates expression of *SLCO1B3*, we first examined the effect of antiandrogens on *SLCO1B3* expression in two well characterized AR-positive prostate cancer cell lines (22Rv1 & LNCaP). Cells were treated with the second-generation antiandrogens abiraterone and enzalutamide, as well as finasteride, for 24 h. In LNCaP cells, abiraterone, enzalutamide and finasteride modestly decreased *SLCO1B3* mRNA levels (Fig. 1a). Enzalutamide had a modest effect on transcript levels in 22Rv1 cells (Fig. 1b). Surprisingly, treatment with 20 µM abiraterone produced a significant increase in *SLCO1B3* expression in 22Rv1 cells at 24 h (Fig. 1b), and this inhibition was sustained up to 72 h of treatment (Supplementary Fig. S1).

Since a subset of patients is inherently resistant to both abiraterone and enzalutamide due to expression of AR-V7^25^, we next examined the mechanism of abiraterone-induced upregulation of *SLCO1B3* expression in 22Rv1 cells as a possible mechanism of resistance since this cell line expresses high levels of endogenous AR-variants (e.g., AR-V7) and exhibits intrinsic resistance to both enzalutamide and abiraterone^26,27^. When cells were treated with varying concentrations of abiraterone, expression of *SLCO1B3* transcript was significantly upregulated in a dose-dependent manner (Fig. 1c). To investigate whether androgen mediated *SLCO1B3* upregulation, we treated 22Rv1 cells in phenol red-free, charcoal-stripped media containing abiraterone in the presence and absence of two androgens, testosterone (T) and dihydrotestosterone (DHT). We found that *SLCO1B3* was significantly upregulated by abiraterone regardless of androgen stimulation (Fig. 1d,e). While the abiraterone + DHT treatment appears to have induced *SLCO1B3* to a lesser extent than ABI treatment alone, there is not a statistically significant difference in the *SLCO1B3* expression levels between two treatment groups. Treatment with T or DHT increased *KLK3* mRNA expression (data not shown), indicating androgen stimulation was potent enough to induce AR-dependent gene expression, but neither increased *SLCO1B3* expression. We also evaluated whether there may be a dose-dependent effect with enzalutamide on *SLCO1B3* transcripts and found that treatment with 10 µM and 20 µM doses of enzalutamide in the presence and absence of DHT did not affect the expression of *SLCO1B3* (Fig. 1e).

Since LNCaP cells express the AR-T878A point mutation and 22Rv1 cells express both full-length and a constitutive active AR splice variant (AR-V7), we next determined the effect of abiraterone on the androgen-responsive VCaP cell line, which harbors the wild-type AR and AR-V7^28^. We showed that abiraterone also increased the expression of *SLCO1B3* mRNA in VCaP cells in a dose-dependent manner similar to that observed in 22Rv1 cells (Fig. 1f).

### Other CYP17A1 inhibitors upregulate SLCO1B3

**Figure 2:**
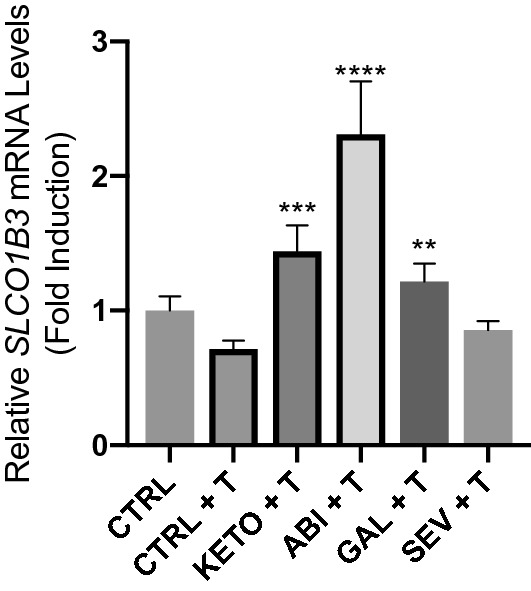
The effect of CYP inhibitors on *SLCO1B3* expression. 22Rv1 cells were treated with four CYP17 inhibitors, ketoconazole (Keto), abiraterone (ABI), galeterone (GAL), or seviteronel (SEV), in media supplemented with 1 nM testosterone for 24 h. Cells were treated with ketoconazole and abiraterone at a concentration of 20 µM and galeterone and seviteronel at a dose of 10 µM based on precedents set for in vitro experiments in previous publications. RNA was extracted and assessed for changes in *SLCO1B3* expression using qPCR. Gene expression was normalized to expression of β-actin. All CYP17 inhibitors but seviteronel produced statistically significant induction of *SLCO1B3* in 22Rv1 cells. Statistical tests were performed against CRTL + T. Data represent three independent experiments with triplicate measurements. **P < 0.001, ***P < 0.0001, ****P < 0.00001.

Since abiraterone is a potent inhibitor of the cytochrome P450 17A1 enzyme (CYP17), we next determined whether other CYP17A1 inhibitors may also be involved in regulating *SLCO1B3* expression. 22Rv1 cells were treated with 20 µM ketoconazole, 10 µM seviteronel, and 10 µM galeterone alongside 20 µM abiraterone to assess whether upregulation of *SLCO1B3* is abiraterone-specific, or whether this effect is common to the CYP17A1 inhibitor drug class. All drugs tested (except seviteronel) induced a statistically significant increase in *SLCO1B3* transcripts, with abiraterone producing the largest increase of *SLCO1B3* expression (Fig. 2).

### Abiraterone upregulates expression of tumor *SLCO1B3* in a 22Rv1 mouse xenograft model

**Figure 3:**
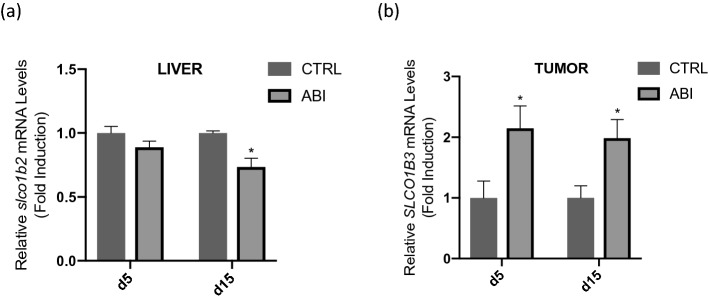
Mice bearing 22Rv1 prostate cancer xenografts were treated with abiraterone acetate (AA) at 5 mmol/kg/day or vehicle control (95% safflower oil, 5% benzyl alcohol) via intraperitoneal injection every day for either (**a**) 5 or (**b**) 15 days. RNA was extracted from harvested tumor or liver tissue and reverse transcribed for analysis by qPCR. Gene expression was normalized to expression of β-actin. Significant upregulation of *SLCO1B3* was observed at both time points. Mouse livers were assessed for changes in *slco1b2* (the mouse analogue of human *SLCO1B3*) expression. While AA treatment induced a statistically significant decrease in mouse liver *slco1b2* expression, the magnitude of the change was small and may not reflect how *SLCO1B3* expression in a human liver would be affected by AA treatment. *P < 0.05.

To assess whether abiraterone could upregulate *SLCO1B3* in vivo, we treated mice bearing 22Rv1 xenografts with abiraterone acetate or vehicle control for either 5 or 15 days. As anticipated abiraterone treatment did not reduce the growth of 22Rv1 xenografts (data not shown), consistent with previous studies^26,29^. After 5 days of abiraterone acetate treatment, tumors were excised and digested to measure mRNA transcripts. Abiraterone-treated tumors exhibited a 2.5-fold increase in *SLCO1B3* mRNA expression when compared with vehicle control (p < 0.05) (Fig. 3a). After 15 days of treatment, abiraterone-treated mice had a similar upregulation of *SLCO1B3* (p < 0.05) when compared with control mice treated with vehicle control. Levels of *slco1b2* (the mouse analogue of human *SLCO1B3*) in the livers of the mice were modestly decreased at day 15 of treatment (Fig. 3b).

### Abiraterone-induced overexpression of *SLCO1B3* is mediated by miRNA expression

**Figure 4:**
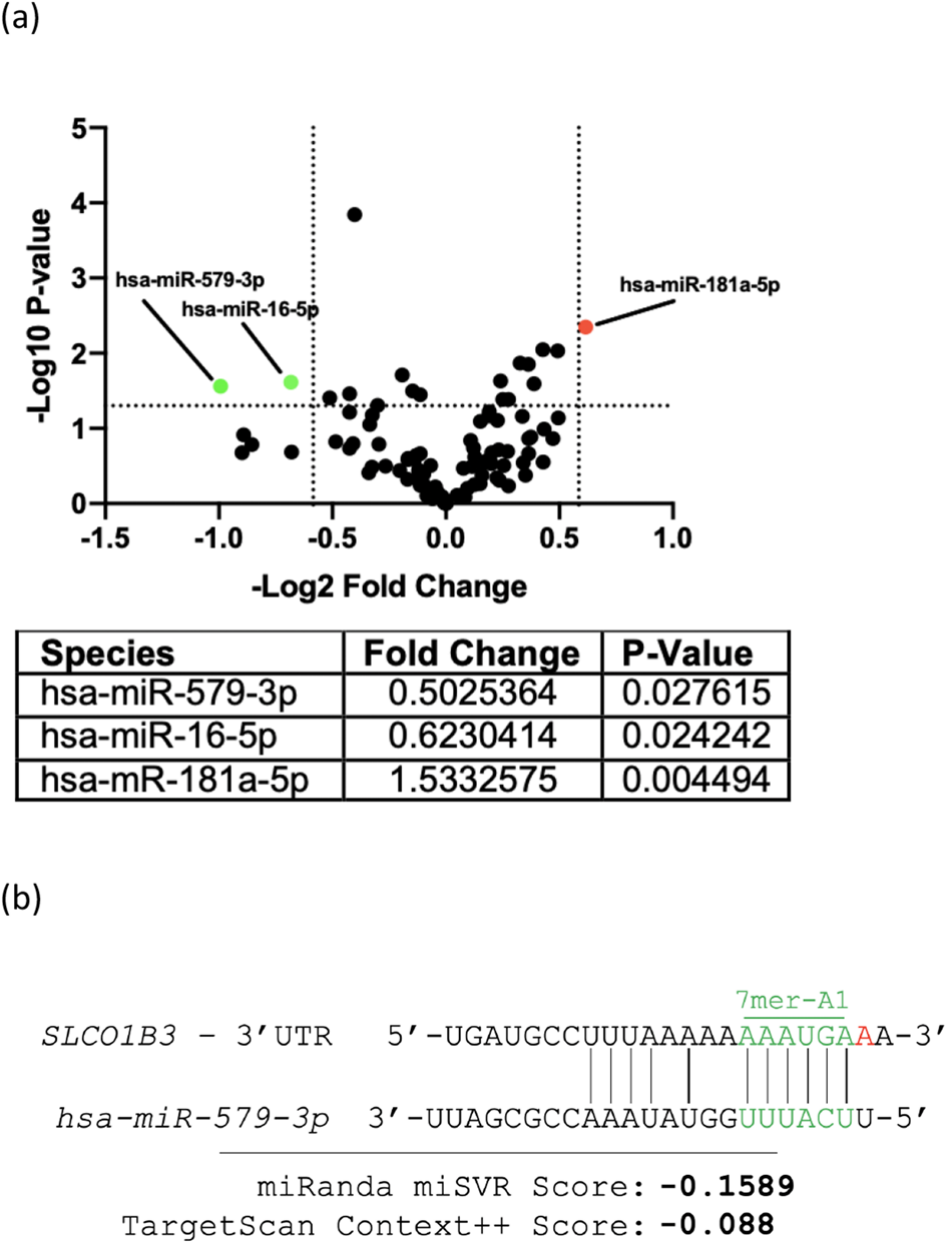
To investigate the relationship between miRNA and *lt-SLCO1B3* expression, we profiled the miRNA transcriptome of cells treated with abiraterone using NanoString. (**a**) Differentially expressed miRNA species above 1.5-fold change and P < 0.05 in an unpaired Student’s T-test were selected for further analysis. (**b**) miRNA–mRNA binding prediction programs miRanda and TargetScan identified binding between hsa-miR-579-3p and *SLCO1B3.* Images show binding of hsa-miR-579-3p to the promoter of SLCO1B3 using TargetScan and miRanda.

We have previously demonstrated that the liver-type *SLCO1B3* (*lt-SLCO1B3*) was predominantly overexpressed in prostate tumor tissues^1^. We next determined whether abiraterone-induced *SLCO1B3* mRNA expression occurred at the promoter level. Analysis of the *SLCO1B3* promoter (containing the full-length 1492 bp upstream promoter from the transcription start site) characterized by luciferase reporter assays showed no cis-regulatory response to abiraterone treatment (Supplementary Fig. S2). We therefore hypothesized that the mechanism driving abiraterone-induced upregulation of *SLCO1B3* was post-transcriptional, likely mediated by miRNA. To investigate the relationship between miRNA and *lt-SLCO1B3* expression, we profiled the miRNA transcriptome of 22Rv1 cells treated with 20 µM abiraterone for 24 h, using the NanoString nCounter Human v3 miRNA panel. This assay rapidly and efficiently profiles 800 highly curated human miRNAs. We chose this hybridization-based nucleic acid counting method to avoid amplification biases, at the expense of resolving low-abundance miRNA species. Three miRNA species hsa-miR-579-3p, hsa-miR-16-5p, and hsa-mir-181a-5p were identified as significantly differentially expressed, demonstrating greater than 1.5-fold change (P<0.05) (Fig. 4a and Supplementary Fig. S3).

To predict miRNA species with stable expression under abiraterone treatment, globally normalized counts from the NanoString panel were input in RefFinder, a program which aggregates stability-ranking programs BestKeeper, NormFinder, Genorm, and the comparative ∆-Ct method by taking the geometric mean of each program’s generated rankings. Hsa-let-7e-5p, hsa-miR-32-5p, and hsa-miR-148b-3p were predicted to be the most stable potential reference genes (Table 1). Stability of these reference genes was validated by qPCR analysis. As it was ranked as most stable by RefFinder, hsa-let-7e-5p was selected as the reference species against which the selected miRNA species would be measured, although use of hsa-miR-32-5p and hsa-miR-148b-3p provided similar results.

**Table 1:** RefFinder ranking of miRNA Species.

Rank	miRNA species	Delta CT	BestKeeper	NormFinder	Genorm	Geomean of ranking
1	hsa-let-7e-5p	1	2	2	1	1.41
2	hsa-miR-32-5p	2	1	1	21	2.55
3	hsa-miR-148b-3p	3	3	3	22	4.94
4	hsa-miR-592	5	5	4	23	7
5	hsa-miR-361-5p	4	4	7	23	7.12
6	hsa-miR-582-5p	6	7	10	25	10.12
7	hsa-miR-9-5p	8	11	6	26	10.82
8	hsa-miR-99a-5p	11	10	9	27	12.79
9	hsa-miR-98-5p	7	9	14	36	13.35
10	hsa-miR-22-3p	9	6	16	36	13.37

MiRNA species identified as significantly differentially expressed by the NanoString panel were then screened by TargetScan3.0^30^ and miRanda software^31^, miRNA-mRNA binding domain alignment programs. Only hsa-miR-579-3p was predicted by TargetScan and miRanda to bind to the 3′-UTR of the *lt-SLCO1B3* transcript (Fig. 4b). The Context++ score generated by TargetScan7.2 is output from a model incorporating 14 miRNA-binding parameters and is reflective of the probability and extent to which an miRNA species will repress expression of a given mRNA target^30^. The Context++ score of − 0.088 given to the hsa-miR-579-3p*/SLCO1B3-*3′UTR is in the 92nd percentile for predicted *SLCO1B3* 3′UTR-binding miRNA species. The miRanda program is a machine learning-based predictive binding algorithm, and the calculated score of − 0.1589 reflects an approximate 40–50% probability that hsa-miR-579-3p represses transcript level of *SLCO1B3* to the extent which we have observed, which is approximately 30–50%^32^.

**Figure 5:**
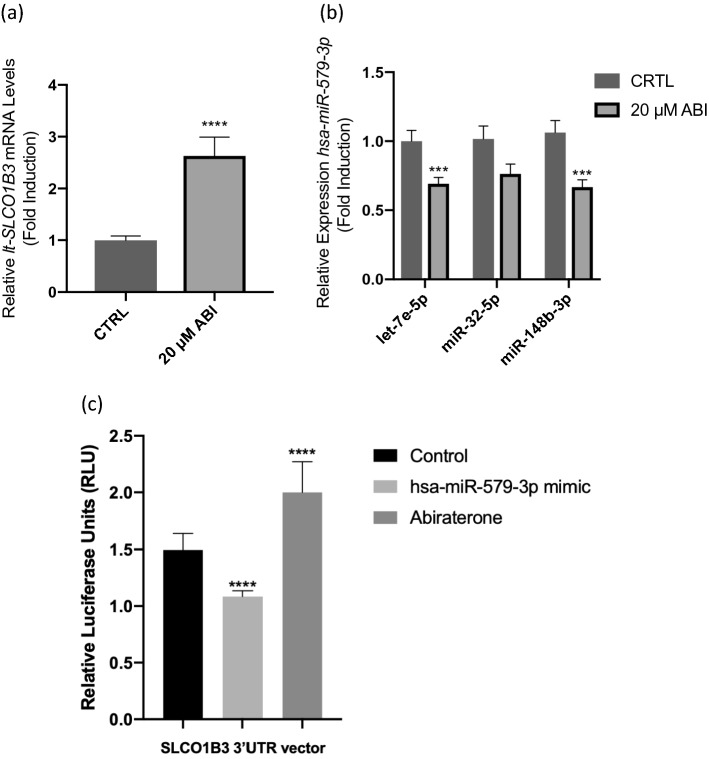
Abiraterone induces overexpression of *lt-SLCO1B3* while suppressing hsa-miR-579-5p expression. Effects of abiraterone treatment on the expression of (**a**) *lt-SLCO1B3* and (**b**) hsa-miR-579-3p in the samples processed for NanoString analysis were validated by qPCR analysis. As expected hsa-miR-579-3p expression was reduced and *lt-SLCO1B3* expression was increased following abiraterone treatment, consistent with miRNA-transcript repression. (**c**) hsa-miR-579-3p directly binds to *SLCO1B3* 3′UTR. Co-transfection of 22Rv1 cells was performed with the 3′UTR reporter plasmid (or vector control plasmid) and hsa-miR-579-3p mimic (or negative control mimic) for 24 h followed by luciferase reporter assays. The assay revealed a significant decrease in *SLCO1B3* reporter activity in the presence of the mimic. 22Rv1 cells were also transfected with the 3′UTR plasmid (or vector control plasmid) followed by treatment with 20 µM Abiraterone (or vehicle DMSO control) for 24 h. Abiraterone treatment resulted in an increase in *SLCO1B3* luciferase reporter activity. No significant change was observed in the vector control plasmid lacking the 3′UTR (see Supplementary Fig. S4). These data are the results of three independent experiments performed in triplicates. ***P < 0.005, ****P < 0.0001.

The effects of abiraterone treatment on the expression of *lt-SLCO1B3* and hsa-miR-579-3p in the samples processed for NanoString analysis were further validated by qPCR. Recapitulating the NanoString results, qPCR analysis found hsa-miR-579-3p miRNA expression to be reduced and *lt-SLCO1B3* mRNA expression increased following abiraterone treatment (Fig. 5a,b). This is consistent with miRNA-mediated repression of *lt-SLCO1B3.* Using luciferase reporter system, we further demonstrated that hsa-miR-579-3p indeed binds to the *SLCO1B3* 3′UTR by cloning this sequence into a psiCHECK-2 vector, containing the renilla and continuously expressed firefly luciferase reporter gene. Next, we performed co-transfections of the psiCHECK-2-*SLCO1B3* 3′UTR plasmid (or empty vector plasmid) together with the hsa-miR-579-3p mimic or nonspecific mimic (negative control) in 22Rv1 cells followed by assaying for luciferase activity. Dual-luciferase reporter assay verified that *SLCO1B3* is a target gene of hsa-miR-579-3p as our data demonstrated a significant decrease in luciferase reporter activity when cells were treated with the hsa-miR-579-3p mimic in comparison to the negative control (Fig. 5c), indicating that hsa-miR-579-3p directly targets *SLCO1B3* by binding to its 3′UTR. We also showed that no significant difference was observed in cells transfected with a psiCHECK-2 vector lacking the 3′-UTR, further confirming the specific hsa-miR-579-3p binding interaction with the 3′UTR of *SLCO1B3* (Supplementary Fig. S4). Luciferase activity was recovered and further increased in cells transfected with the psiCHECK-2-SLCO1B3 3′ UTR plasmid and treated with abiraterone as compared to the vehicle DMSO treated control, demonstrating that abiraterone treatment decreased hsa-miR-579-3p miRNA expression, resulting in increased *SLCO1B3* reporter activity (Fig. 5c and Supplementary Fig. S4). These results directly support our findings from both NanoString and qPCR analyses that hsa-miR-579-3p binds to the *SLCO1B3* 3′UTR and provide additional evidence for a mechanism of abiraterone-induced *SLCO1B3* expression mediated by hsa-miR-579-3p.

## Discussion

We have shown that abiraterone, one of the most commonly prescribed drugs for CRPC, induces a significant upregulation of *SLCO1B3* transcripts in 22Rv1 and VCaP cells and in mice bearing 22Rv1 tumor xenografts. Expression levels of *total-SLCO1B3* expression increase with abiraterone treatment in a dose-dependent manner in both 22Rv1 and VCaP cells. We demonstrated that abiraterone-mediated increase in *SLCO1B3* transcripts occurred independently of androgen stimulation, consistent with our finding that abiraterone treatment in the androgen-independent PC3 cell line had no effect on *SLCO1B3* expression (data not shown). We have previously shown that PC3 cells have higher endogenous expression of *SLCO1B3* than 22Rv1 cells^1^ and have low level expressions of the CYP17A1 enzyme; therefore, these cells may not be as responsive to the effect of abiraterone treatment^33^.

We further demonstrated that other CYP17A1 inhibitors (abiraterone, ketoconazole, and galeterone) can upregulate *SLCO1B3* transcripts. Abiraterone, ketoconazole, and galeterone inhibit both 17a-hydroxylase and 17,20-lyase of CYP17A1 whereas seviteronel has selective inhibition of 17,20-lyase over 17a-hydroxylase^34,35^. Whether 17,20-lyase selectivity or potency can explain why seviteronel does not affect *SLCO1B3* remains to be determined^36^.

We showed that the mechanism of abiraterone-induced *SLCO1B3* expression is regulated by miRNAs (hsa-miR-579-3p, hsa-miR-16-5p, and hsa-mir-181a-5p*)*. We used two different miRNA target prediction tools to determine which of the three miRNAs interact with *SLCO1B3.* Using this computational approach, we predicted binding of hsa-miR-579-3p to the 3′-UTR of the *SLCO1B3*. Validation experiments were subsequently performed to confirm the NanoString data and revealed that the increase in abiraterone-mediated *SLCO1B3* transcripts correlated with a decrease in expression of hsa-miR-579-3p, as verified with luciferase reporter assays demonstrating the binding of hsa-miR-579-3p to the *SLCO1B3* 3′UTR. Hsa-miR-579-3p has previously been shown to be downregulated in exosomes secreted by prostate cancer cells under hypoxic conditions, and is otherwise implicated in tumorigenesis in multiple other cell types via modulation of oncogenic kinase signaling pathways, such as PI3K, BRAF, RAS, and AKT^37–40^.

Previous studies have reported that *SLCO1B3* can be regulated by miRNAs. In a study evaluating membrane drug transporters and miRNA gene expression changes mediated by rifampin treatment in hepatocytes, *SLCO1B3* expression was reduced after rifampin treatment and negatively correlated with rifampin-induced hsa-miR-92a expression^18^. In addition, hsa-miR-192 was found to inhibit FXR, a known regulator of *SLCO1B3*, which in turn, suppressed *SLCO1B3* expression^19,41^.

A recent study by Zedan et al. found hsa-miR-141-3p and hsa-miR-375-3p to be of clinical significance in predicting the outcome of patients treated with abiraterone. The study findings were based on analysis of a pre-selected group of five miRNAs that came from a literature review rather than using a global miRNA profiling approach like NanoString; thus, this may explain the discrepancy in their results, in addition to patient heterogeneity and exposure to prior treatment regimens^42^. Our inability to detect significant differential expression of these species is likely due to the difference in model systems, where we used an in vitro cell line model (a limitation of our study) rather than patient samples. Future studies using clinical samples would be needed to confirm whether hsa-miR-579-3p can be identified in patients treated with abiraterone and to correlate this observation with *SLCO1B3* expression. It also remains to be determined the effect of abiraterone-induced *SLCO1B3* expression on tissue abiraterone levels since the drug has been hypothesized to be an SLCO substrate^43^. Our study is further limited by the use of 22Rv1 cells with intrinsic resistance to abiraterone; future studies should examine the effect of miRNA-mediated *SLCO1B3* expression in cell lines with acquired resistance to the drug as well as subsequent transport of substrates.

Our study identified two miRNAs, hsa-miR-16-5p and hsa-mir-181a-5p, that were differentially regulated by abiraterone treatment. Both miRNAs have previously been identified as potential diagnostic and therapeutic biomarkers for prostate cancer^44–49^. Interestingly, hsa-miR-181a-5p is known to suppress GRP78 in 22Rv1 cells^50^. GRP78, coordinates with SIAH2 to degrade AR-V7^51^. Induction of hsa-miR-181a-5p could therefore be another miRNA-mediated mechanism of resistance against abiraterone. Therefore, while these two miRNAs may not be involved in regulating *SLCO1B3* as a potential mechanism for abiraterone resistance, they may contribute to the overall abiraterone resistance mechanism and future studies are warranted to investigate their relevance.

In summary, we have shown that CYP17 inhibitors can stimulate the expression of *SLCO1B3*, the known testosterone uptake transporter. We demonstrated that the mechanism of abiraterone-induced *SLCO1B3* expression is mediated by the miRNA, hsa-miR-579-3p. To our knowledge, this is the first report of a mechanism of resistance of abiraterone treatment attributed to miRNA regulation in controlling the expression of OATP1B3, an androgen transporter that has been implicated in driving the resistance to ADT through the mechanism of increasing uptake of residual androgens into prostate tumors. In addition, hsa-miR-579-3p may serve as a potential therapeutic biomarker for abiraterone resistance.

## Materials and methods

### Cell culture

All prostate cancer cell lines (CWR22Rv1, LNCaP, PC3, and VCaP) were purchased from ATCC (Manassas, VA). Cell culture reagents were obtained from Gibco/Thermo Fisher Scientific (Gaithersburgh, MD), unless otherwise specified. The 22Rv1 and LNCaP cell lines were maintained in phenol red-free RPMI 1640 Medium supplemented with 10% fetal bovine serum (FBS, Atlanta Biologicals, Flowery Branch, GA), 100 units/mL penicillin, 100 units/mL streptomycin, and 0.25 μg/mL amphotericin B. For all experiments involving addition of steroid hormone, cells were plated in maintenance media, then medium was replaced with RPMI Medium 1640 supplemented with 10% charcoal stripped FBS (all other media components remained unchanged) for approximately 7 h before treatment. PC3 cells were cultured in F-12K Nutrient Mixture medium (Gibco), and VCaP cells were maintained and treated in DMEM Medium (Gibco) supplemented with 10% FBS, 100 units/mL penicillin, 100 units/mL streptomycin, and 0.25 μg/mL amphotericin B.

### Reagents

Abiraterone and enzalutamide were purchased from Selleckchem (Houston, TX). Galeterone, finasteride, and ketoconazole were purchased from Sigma-Aldrich (St. Louis, MO). VT-646 (seviteronel) was ordered from Chemscene (Monmouth Junction, NJ). Abiraterone acetate for xenograft studies was purchased from Medkoo Biosciences (Morrisville, NC). All drugs were dissolved in DMSO, aliquoted and stored at − 80 °C. Testosterone (Sigma) was dissolved in ethanol and DHT (Steraloids Inc., Newport, RI) was dissolved in DMSO.

### Semiquantitative real-time polymerase chain reaction

22RV1 and LNCaP cells were plated in 6-well dishes in maintenance media the day before treatment. Cells were serum-starved approximately 7 h prior to treatment and then incubated with the treatment medium for 24 h or 72 h. Total RNA was extracted from cells using the QIAshredder and RNeasy mini kits (Qiagen) as per the manufacturer’s protocol. Extracted RNA was then assessed for purity using a NanoDrop spectrophotometer (Molecular Devices) and all samples were diluted to a concentration of 80 ng/µL in RNase-free water. Using 12 µL of each of these stocks, cDNA was synthesized using the SuperScript III First-Strand Synthesis System for RT PCR (Thermofisher) as per the manufacturer’s protocol.

The RT PCR reaction yields 30 µL of cDNA reaction product. Two microliters of this cDNA was amplified by qPCR using the following TaqMan probes: SLCO1B3 (Hs00251986_m1, Applied Biosystems), LT-SLCO1B3 (Hs01127179_mH, Applied Biosystems). For each qPCR reaction, 2 µL of cDNA was mixed with 10 µL of Taqman Gene Expression Master Mix (Applied Biosystems), 7 µL of RNAse-free water, and 1 µL of the respective Taqman qPCR primer. Semiquantitative Real-Time Polymerase Chain Reaction (qPCR) was performed using an Applied Biosystems StepOnePlus Real-Time PCR system with StepOne Software. Each qPCR reaction was run in triplicate and gene expression was normalized to expression of β-actin. Fold-change in gene expression was calculated using the ΔΔC_t_ method as described in the SABiosciences 2009 RT2 Profiler PCR Array System User Manual (SABiosciences, Frederick, MD).

### Prostate cancer xenograft study

Six-week old, male, severe combined immunodeficiency (SCID) mice were obtained from the NCI-Frederick Animal Production Area. 22Rv1 cells were cultured in maintenance media and harvested when they reached 80% confluency. Cells were washed with sterile phosphate buffered saline (Gibco) and approximately 6 million cells were injected subcutaneously into the rear flank of each SCID mouse. Mice were monitored and weighed daily. When tumor volumes reached ~ 200–250 mm^3^, animals were randomized into two groups of control vs treatment (n = 5). Each group was treated daily with intraperitoneal (i.p.) bolus injections of either the drug vehicle (95% safflower oil, 5% benzyl alcohol) or abiraterone acetate (0.5 mmol/kg) for either 5 or 15 consecutive days^52^. Tumor measurements used to calculate tumor volume (tumor volume = tumor length*tumor width*tumor height*π/6) were taken three days per week and mice were weighed daily. Mouse livers and tumors were excised on either day 5 or day 15 of treatment, and harvested tissue samples were snap frozen in liquid nitrogen for subsequent gene expression analysis.

To analyze gene expression in the liver and tumor tissues, tissue was thawed on ice and an approximately 30 mg section was shaved off using a scalpel. This section was placed in a bead homogenizer tube with 600 µL of buffer RLT (Qiagen, Hilden, Germany) and homogenized for roughly 30 s (BeadBug Benchtop Homogenizer, Benchmark Scientific). After the tissue was homogenized in lysis buffer, an equal (600 µL) volume of 70% alcohol was added to the tube and mixed gently to precipitate RNA. Then, 700 µL of the precipitated RNA mixture was transferred to an RNeasy Mini spin column (Qiagen) and the RNA extraction proceeded according to the RNeasy Mini Kit protocol (Qiagen). RNA was reverse-transcribed to cDNA and used for qPCR analysis using Mouse ACTB (4352663, Applied Biosciences) and *slco1b2* (Mm00451510_m1, Applied Biosystems) probes. qPCR and data analysis were performed as previously described.

The National Cancer Institute (NCI) is accredited by the Association for Assessment and Accreditation of Laboratory Animal Care (AAALAC) International and follows the Public Health Service (PHS) Policy for the Care and Use of Laboratory Animals. Animal care was provided in accordance with the Guide for the Care and Use of Laboratory Animals. The study protocol was approved by the NCI Animal Care and Use Committee. This study was carried out in compliance with the ARRIVE guidelines.

### miRNA analysis

#### miRNA extraction

22Rv1 cells were homogenized using a QIAshredder kit (Qiagen Cat#: 79654) and total RNA was extracted from the homogenate with a miRNeasy Mini Kit (Qiagen Cat#: 217004) following manufacturer protocols. RNA was quantified for normalization using a NanoDrop ND-1000 spectrophotometer (Thermo Scientific).

#### miRNA profiling

Total RNA was prepared for and profiled using the nCounter Human v3 miRNA Panel (NanoString Technologies) on the nCounter Analysis System (NanoString Technologies) according to the manufacturer’s protocol. Total RNA was loaded at 100 ng per sample, hybridizations were 17–22 h long, and counts were gathered by scanning on HIGH mode for 280 fields of view per sample. Normalization and analysis of the NanoString panel data were performed using nSolver Software (NanoString Technologies). Base threshold was set to 20 counts. Global count normalization was performed using the geometric mean method as described in the nCounter Expression Data Analysis Guide (NanoString Technologies MAN-C0011-02). Linear ratio of normalized miRNA panel values was calculated by dividing the geometric mean of each experimental group by the geometric mean of the control, following the method described in the Gene Expression Data Analysis Guidelines (NanoString Technologies MAN-C0011-04). For each miRNA species, Log2 of each abiraterone and vehicle control value divided by the geometric mean of the vehicle control was calculated. The paired Student’s t-test was used to identify significant change in miRNA ratio. The Log2 fold-change values were plotted against the − Log10 of the P-value for each miRNA species to generate the volcano plot.

#### miRNA qPCR

MiRNA relative expression was quantified with TaqMan Advanced miRNA assays (Thermofisher). Normalized NanoString panel data, containing the top 100 most highly expressed species, was input into RefFinder^53^ to identify relatively highly expressed stable reference miRNA species: hsa-let-7e-5p, hsa-miR-32-5p, and hsa-miR-148b-3p. cDNA synthesis was performed using the Taqman Advanced miRNA Synthesis Kit (Thermofisher, Cat#: A28007) following the manufacturers protocol with a 10 ng total RNA input. qPCR was performed following manufacturer’s instructions using TaqMan Fast Advanced MasterMix (Thermofisher Cat#: 4444556) and Taqman Advanced miRNA Assays: hsa-let-7e-5p (Cat#: 478579_miR), hsa-miR-32-5p (Cat#: 478026_miR), hsa-miR-148b-3p (Cat#: 477824_miR), and hsa-miR-579-3p (Cat#: 479059_miR). Relative miRNA expression was calculated using the 2^−∆∆Ct^ method, comparing hsa-miR-579-3p Ct to the corresponding mean of the stable reference miRNA species.

### Construction of *SLCO1B3* 3′UTR luciferase reporter plasmid and reporter assays

The *SLCO1B3* 3′UTR was synthesized with flanking *NotI* and *XhoI* restriction sites in a pUC57 vector by GENEWIZ, Inc (South Plainfield, NJ). The synthesized sequence was then cloned into complementary restriction sites in a psiCHECK-2 vector, which contains both renilla and firefly luciferase reporter genes (Promega, CAT#: C8021). The constructed plasmid was sequenced to verify successful cloning.

22Rv1 cells were seeded in a 96-well dish for 24 h before transfection with 100 ng of psiCHECK-2 empty vector or 3′UTR plasmid using Lipofectamine 2000 (Life Technologies, Cat#: 11668019). The following day, cells were treated with 20 µM abiraterone (SelleckChem, Cat#: S1123) or vehicle control (0.4% DMSO) (Sigma Aldrich). After a 24-h treatment period, samples were lysed and assayed using the Dual-Luciferase Reporter Assay System (Promega, Cat#: E1910) per manufacturer’s protocol. Raw luminescence was measured for Renilla and firefly reporters using SpectraMax iD3 (Molecular Devices). For microRNA mimic treated samples, cells were instead co-transfected for 24 h with the psiCHECK-2 plasmid or empty vector and 1 pmol of hsa-miR-579-3p miRvana mimic or negative control (Life Technologies, Cat#: 4464066) before performing the reporter assay.

### Statistical considerations

The Mann–Whitney U test was performed to test for differences in gene expression when multiple different drug treatments were performed. To assess the significance drug treatment gradients and drug class effects, Dunnett’s multiple comparison test was performed. Mann–Whitney U test was used to determine significance of changes in relative expression levels of hsa-miR-579-3p. All comparisons were conducted using GraphPad Prism software (GraphPad Prism version 6.00, GraphPad Software, La Jolla CA).

## Supplementary Information

Supplementary Information.
