# Supplementary material for: Abiraterone induces SLCO1B3 expression in prostate cancer via microRNA-579-3p
Source: Sci Rep. 2021 May 24;11:10765. doi: 10.1038/s41598-021-90143-4 (PMC8144422; doi:10.1038/s41598-021-90143-4)
Supplement: Supplementary file 1 — Supplementary Information. [file 41598_2021_90143_MOESM1_ESM.docx]

Supplementary Figures

**Manuscript Submission ID**: 8ebd26dd-c73c-4ded-a373-a6f7c5c064db

**Manuscript Title**: “Abiraterone Induces *SLCO1B3* Expression in Prostate Cancer via MicroRNA-579-3p”

**Authors:** Roberto H. Barbier, Edel M. McCrea, Kristi Y. Lee, Jonathan D. Strope, Emily N. Risdon, Douglas K. Price, Cindy H. Chau, William D. Figg

|  |
| --- |

**Supplementary Figure S1.** The AR-positive prostate cancer 22Rv1 cell line was treated with 1nM testosterone and three prostate cancer drugs abiraterone (ABI), enzalutamide (ENZ), or finasteride (FIN) for 72hrs. RNA was extracted and assessed for changes in *SLCO1B3* expression using qPCR. Gene expression was normalized to expression of 𝜷-actin. Abiraterone produced a highly significant upregulation of *SLCO1B3* in 22Rv1 cells over 72hrs.These data are the result of three independent experiments. ***P<0.0001

|  |
| --- |

**Supplementary Figure S2.** The lack of *SLCO1B3* promoter activity following in vitro abiraterone treatment indicated that the induction of *SLCO1B3* by abiraterone was not cis-regulated. A luciferase reporter containing the 1500bp upstream promoter of *SLCO1B3* was lipofected into 22Rv1, PC3, and LNCaP cells and relative luciferase activity was found. Results are reported in fold-change of relative luciferase units relative to the promoterless luciferase plasmid control. These data are the result of three independent experiments.

|  |
| --- |

Supplementary Figure S3. NanoString quantitation of miRNA abundance, shown in Log10 globally-normalized counts. MicroRNA species were arranged in descending fold-change of abundance. Highlighted in red, microRNA species hsa-miR-579-3p, hsa-miR-16-5p, and hsa-miR-181a-5p, are among the most differentially expressed following treatment with 20µM abiraterone for 24hrs.


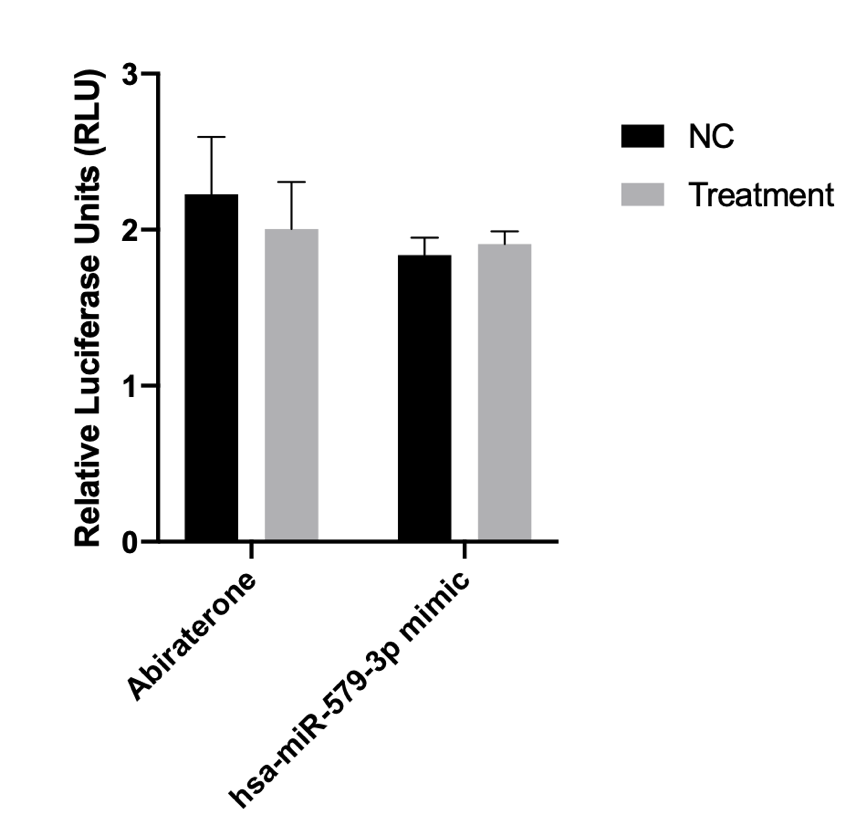


**Supplementary Figure S4**. No significant difference was observed in 22Rv1 cells co-transfected with a psiCHECK-2 vector lacking the 3’-UTR and either hsa-miR-579-3p or nonspecific mimic. Cells were also transfected with psiCHECK-2 empty followed by treatment with abiraterone or vehicle (DMSO) control for 24 hours. These data are the result of three independent experiments.

Supplementary Methods

22Rv1, PC3, and LNCaP cells were plated in 96-well plates in maintenance media the 24 hours prior to transfection. Maintenance media was exchanged for Opti-MEM reduced serum medium (Gibco), supplemented with *SLCO1B3* promoter plasmids. 22Rv1 and PC3 cells were transfected with Attractene purchased from Qiagen (Gaithersburg, MD). LNCaP cells were transfected with Lipofectamine 2000 purchased from ThermoFisher Scientific (Waltham, MA) according to the manufacturer’s recommendations. All cell lines were transfected with 100 ng of Promega pGL4.11[luc2P] (Madison, Wi) containing the full-length 1492 bp upstream promoter from the transcription start site of *SLCO1B3*. Luciferase activity was normalized for transfection efficiency with 1 ng of pGL4.74[hRluc/TK] plasmid from Promega. Transfections were performed following the manufacturer’s recommendation.

To investigate the effects of different drug treatments on luciferase activity, 24hrs post-transfection the Optimem transfection medium was replaced with maintenance media containing 20µM Abiraterone. Cells were incubated with drug treatments for 24hrs, at which point they were assayed for luciferase activity using the Promega DLR (Dual Luciferase Reporter Assay) system. Luciferase activity was quantified using a Promega GloMax-Multi+ Detection System and normalized using the Renilla luciferase measurements.
